# Inhibitory Effect of Astaxanthin on Testosterone-Induced Benign Prostatic Hyperplasia in Rats

**DOI:** 10.3390/md19120652

**Published:** 2021-11-23

**Authors:** Liping Wang, Yiwen Hou, Rong Wang, Qi Pan, Debao Li, Han Yan, Zuyue Sun

**Affiliations:** NHC Key Lab of Reproduction Regulation (Shanghai Institute for Biomedical and Pharmaceutical Technologies), Fudan University, Shanghai 200032, China; lpwang66@163.com (L.W.); m18850542674@163.com (Y.H.); luhequ@163.com (R.W.); panqi2007@163.com (Q.P.); lidebao19960825@163.com (D.L.); yanhan617617@126.com (H.Y.)

**Keywords:** astaxanthin, benign prostatic hyperplasia, pharmacodynamics, SOD activity

## Abstract

This study investigates the inhibitory effect of astaxanthin (AST) on testosterone-induced benign prostatic hyperplasia (BPH) in rats. Except for the sham operation, BPH model rats were randomly assigned to five groups: the BPH model control rats, AST-treated BPH model rats (20 mg/kg, 40 mg/kg, and 80 mg/kg), and epristeride (EPR)-treated BPH model rats. After treatment, as compared with the BPH model control rats, the prostate and ventral prostate weights of the AST-treated rats decreased, while there was a marked decline in the 80 mg/kg AST-treated rats. The same effect was also observed in the prostate index and ventral prostate index. The proliferation characteristics of epithelia observed in the BPH model control group were gradually alleviated in the AST-treated rats. As compared with the BPH model control rats, lower epithelial thicknesses of prostates and fewer secretory granules in epithelia were observed in the AST-treated rats. The superoxide dismutase (SOD) activity of prostates increased in all the AST-treated rats with a significant increase in the 40 mg/kg and 80 mg/kg AST-treated rats. The testosterone (T) and dihydrotestosterone (DHT) levels of prostates in the AST-treated groups were lower than those in the BPH model control group, and a significant decline was found in the T level of prostates in the 40 g/kg and 80 mg/kg AST-treated rats and the DHT level of prostates in the 40 mg/kg AST-treated rats. These results indicate that AST might have an inhibitory effect on T-induced BPH in rats, possibly due to SOD activity regulation and T and DHT levels.

## 1. Introduction

Benign prostatic hyperplasia (BPH) refers to the progressive enlargement of the prostate gland and is one of the most common diseases in the aging male population. Approximately, half of men over 50 years of age develop BPH [1]. BPH is usually accompanied by lower urinary tract symptoms and related complications such as urinary frequency, urinary retention, and urinary tract infection, which may lead to a decrease in the quality of life and even cause extreme annoyance and embarrassment. BPH is typically treated with pharmacological intervention or surgery [2].

Notwithstanding, the first treatment option for many BPH patients is still pharmacological therapies, among which the main classes of medicine include 5α-reductase inhibitors and α-adrenergic blockers are currently used for symptom relief [3]. However, the adverse side effects of these traditional medications, such as retrograde ejaculation, orthostatic hypotension, and erectile dysfunction, which affects many patients, remain a major challenge [4]. Therefore, it is necessary to search for and use “safer” and effective alternatives against BPH.

**Figure 1:**
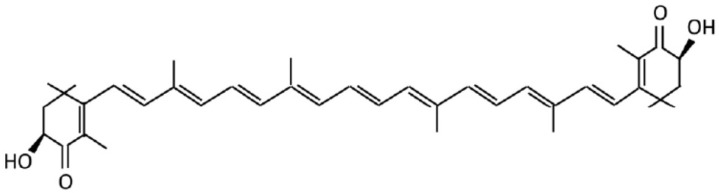
Chemical structure of astaxanthin [7].

Carotenoid astaxanthin (AST) is a red-orange pigment primarily found in organisms, such as shrimp, algae, fish, and crustaceans, containing two oxygenated ends on each tail of the structure (Figure 1) [5,6]. This natural dietary carotenoid has a variety of functions, including antioxidant, anti-inflammatory, and antitumor properties [7,8,9,10]. Moreover, a previous study demonstrated that AST might be safe and well tolerated in rats [11].

Rat prostate mainly consists of paired ventral lobes and paired dorsal lobes [12]. Each lobe is composed of acini, lined by a single epithelium layer and separated by connective tissues containing stromal cells [12,13]. Prostate growth may be stimulated by the continued presence of certain hormones such as testosterone (T) [14]. Dihydrotestosterone (DHT), which is the most active and principal androgen in the prostate, is synthesized from T by the enzyme 5α-reductase. Therefore, the activity of enzyme 5α-reductase, whose isoform 2 predominates in the prostate, may play an important role in the pathogenesis and progression of BPH. Research has suggested that low levels of AST can inhibit 5α-reductase significantly in vitro [15].

Moreover, AST may have an inhibitory effect on prostate cancer. Studies have indicated that AST can modulate the proliferation and apoptosis of cells and inhibit PC-3 xenograft prostate tumors in nude mice [10]. Research has also shown that AST can inhibit the proliferation of aggressive PCa DU145 cells [16].

However, to the best of our knowledge, there is no evidence of the efficacy of AST on BPH in vivo. In the present study, we investigated whether AST could inhibit the development of T-induced BPH in rats.

## 2. Results

### 2.1. Effect of AST on Prostate Weight

**Figure 2:**
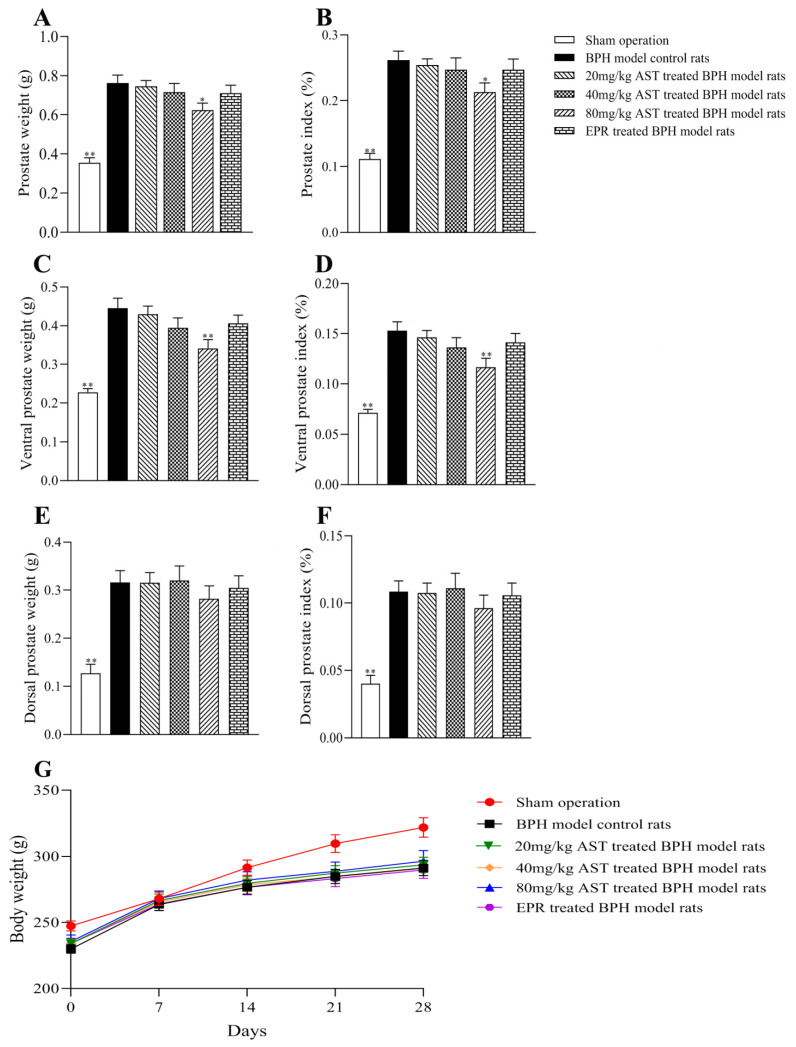
Rat body weights (**G**) and the effect of AST on the weight of the prostate and the prostate index (including ventral and dorsal prostate). Data represent the mean ± SEM (*n* = 12). * Significantly different from BPH model control group, *p* < 0.05 and ** significantly different from BPH model control group, *p* < 0.01. (**A**) Prostate weight; (**B**) prostate index; (**C**) ventral prostate weight; (**D**) ventral prostate index; (**E**) dorsal prostate weight; (**F**) dorsal prostate index.

T was used to induce prostatic hyperplasia in rats for four weeks, and the prostate (including ventral and dorsal prostate) weight of each rat was measured. As shown in Figure 2A,C, as compared with the BPH model control rats, the prostate and ventral prostate weights of the AST-treated rats decreased in a dose-response manner, while the prostate weights (*p* < 0.05) and ventral prostate weights (*p* < 0.01) of the 80 mg/kg AST-treated rats significantly declined. Prostate weights and ventral prostate weights of EPR-treated rats were also lower than those of the BPH model control rats. Dorsal prostate weights were not significantly different between the treated groups and the BPH model control group, as shown in Figure 2E.

### 2.2. Effect of AST on the Body Weight and Prostate Index

The body weights of rats treated with AST or epristeride (EPR) for four weeks are shown in Figure 2G. The prostate index was defined as the ratio of prostate weight to the rat’s body weight. The same effects shown in the prostate weights and ventral prostate weights were also found in the prostate index and ventral prostate index values in the AST-treated and EPR-treated rats (Figure 2B,D). The dorsal prostate index values in the treated groups and the BPH model control group did not differ significantly, as shown in Figure 2F.

### 2.3. Effect of AST on the Histopathology and Ultrastructural Pathology of Prostate Tissues

**Figure 3:**
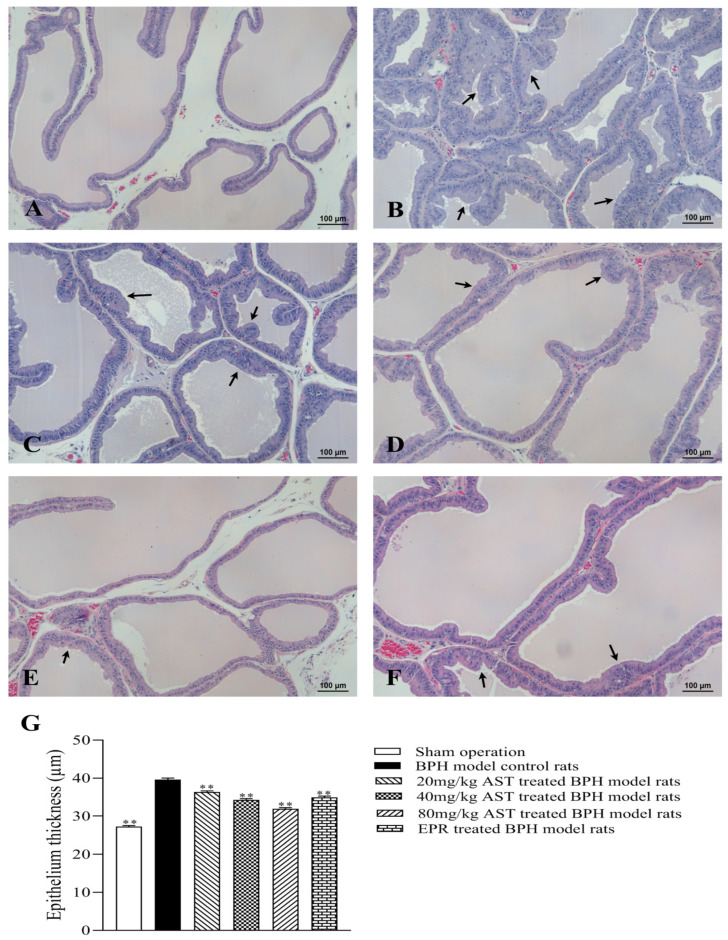
Effect of AST on histomorphology and epithelial thicknesses of ventral prostates in BPH rats. (**A**) No abnormal histological changes were observed in the sham operation; tall columnar epithelium, simple multifocal epithelial thickening with cellular crowding, and folding of the lining epithelium extending into lumina (arrows) were observed in: (**B**) the BPH model control rats; (**C**) 20 mg/kg, (**D**) 40 mg/kg, and (**E**) 80 mg/kg AST-treated BPH rats; (**F**) EPR-treated BPH rats; Hematoxylin and eosin (H&E) 100×; (**G**) epithelial thicknesses of prostates in the sham operation, BPH model control group, and AST or EPR treated groups. Data represent the mean ± SEM. (*n* = 12). ** Significantly different from BPH model control group, *p* < 0.01.

A histopathological examination, called the “golden standard,” is one of the most important and reliable diagnostic methods for the evaluation of pathogenesis. The effect of AST on the histomorphology of ventral prostates in BPH model rats is shown in Figure 3A–F. The histological features found in the BPH model control rats were tall columnar epithelium, simple multifocal epithelial thickening with cellular crowding, and folding of the lining epithelium extending into lumina (Figure 3B). As compared with the BPH model control rats, these features were gradually ameliorated in the AST-treated rats (Figure 3C–E) in a dose-dependent manner, while similar morphological changes were observed in the EPR-treated rats (Figure 3F).

The epithelial thicknesses of ventral prostates in all rats were measured, and the average values of epithelial thickness in all groups are shown in Figure 3G. The average values of epithelial thickness in the AST-treated rats were significantly lower (*p* < 0.01) than in the BPH model control rats, shown in a dose-response manner; the average values of epithelial thickness in the EPR-treated rats also markedly decreased as compared with those in the BPH model control rats (*p* < 0.01).

**Figure 4:**
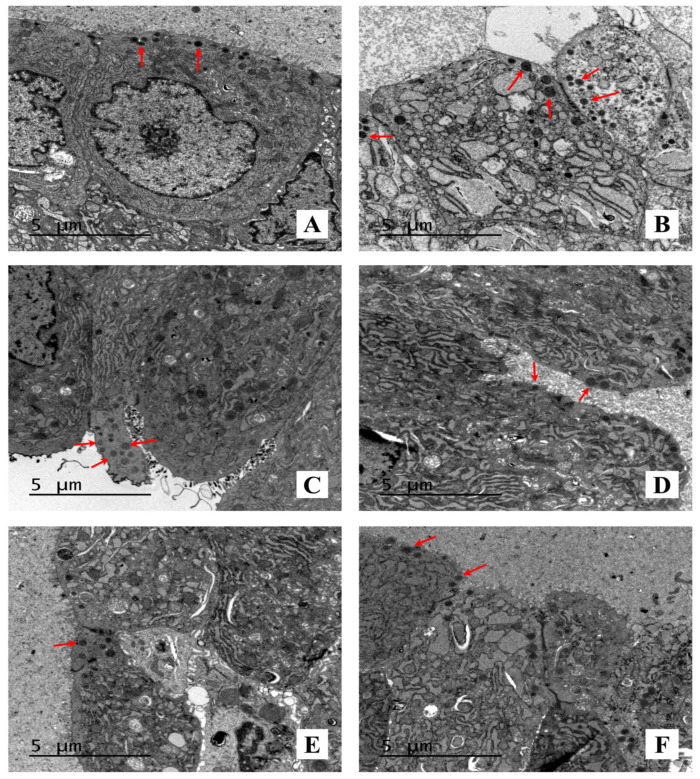
Images of ventral prostates under TEM, 4200×. Secretory granules (arrows) were observed in: (**A**) Sham operation; (**B**) BPH model control rats; (**C**) 20 mg/kg, (**D**) 40 mg/kg, and (**E**) 80 mg/kg AST-treated BPH rats; (**F**) EPR-treated BPH rats.

Secretory granules in ventral prostates were observed using transmission electron microscopy (TEM), as shown in Figure 4. As compared with the BPH model control rats, there were fewer secretory granules observed in the AST-treated rats in a dose-dependent manner.

### 2.4. Effect of AST on the Superoxide Dismutase (SOD) Activity of Prostates

**Figure 5:**
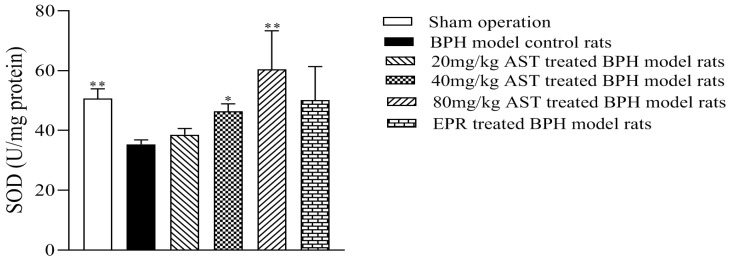
AST increased the SOD activity of ventral prostates. Data represent the mean ± SEM. (*n* = 5). * Significantly different from BPH model control group, *p* < 0.05, and ** significantly different from BPH model control group, *p* < 0.01.

SOD is an important antioxidant enzyme in organisms. The effect of AST on the SOD activity of ventral prostates in the BPH model rats is shown in Figure 5. As compared with the BPH model control rats, the SOD activity of prostates in the AST-treated rats increased in a dose-dependent manner, while the SOD activity of prostates in the 40 mg/kg (*p* < 0.05) and 80 mg/kg (*p* < 0.01) AST-treated rats increased significantly.

### 2.5. Effect of AST on the Levels of T and DHT

**Figure 6:**
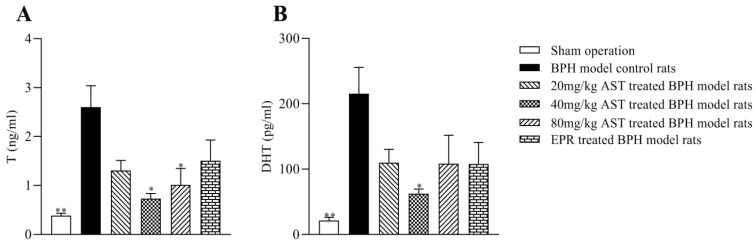
AST decreased levels of T (**A**) and DHT (**B**) in ventral prostates. Data represent the mean ± SEM. (*n* = 5). * Significantly different from BPH model control group, *p* < 0.05, and ** significantly different from BPH model control group, *p* < 0.01.

T and DHT are the primary androgens in the prostate. The levels of T and DHT in ventral prostates in all groups are shown in Figure 6A,B. As compared with the BPH model control rats, the prostate T and DHT levels in the AST-treated rats decreased, while the T levels in the 40 mg/kg and 80 mg/kg AST-treated rats significantly decreased (*p* < 0.05); the DHT levels of prostates in the 40 mg/kg AST-treated rats (*p* < 0.05) also decreased significantly.

## 3. Discussion

To the best of our knowledge, this is the first study to evaluate the inhibitory effect of AST on BPH in vivo. BPH is characterized by an enlarged prostate. Castration was performed to eliminate the influence of endogenous androgen, and rats were given a 5 mg/kg BW/day dose of exogenous androgen to build the BPH model, considered to be relatively closer to the pathogenesis of clinical BPH, as previously reported [17,18]. In the present study, except for the sham-operated rats, BPH model rats were randomly divided into five groups: BPH model control rats, AST (20 mg/kg, 40 mg/kg, and 80 mg/kg)-treated BPH model control rats, and EPR-treated BPH model control rats. A decline in the prostate and ventral prostate weights and index of prostates and ventral prostates in the AST-treated groups were observed, and a significant decline was found in the 80 mg/kg AST-treated group as compared with the BPH model control rats. The prostate and ventral prostate weights and the prostate index values of prostates and ventral prostates in the 80 mg/kg AST-treated rats were also lower than those in the EPR-treated rats. However, the dorsal prostate weights and dorsal prostate index values of the AST-treated rats did not differ significantly from those of the BPH model control rats. Hence, the results suggest that AST might have a more significant inhibitory effect on ventral prostates than dorsal prostates in T-induced rats.

Histological observation of BPH is described as the proliferation of the stroma and epithelia. After treatment for four weeks, the histomorphology of prostates in the T-induced BPH rats was evaluated. The proliferation characteristics of epithelia such as tall columnar epithelia, simple multifocal epithelial thickening with cellular crowding, and folding of the lining epithelia extending into the lumina were markedly observed in the BPH model control group, which were gradually alleviated following AST treatment. The epithelial thicknesses of prostates in all rats were measured, and secretory granules in the epithelia were also observed under TEM. The epithelial thickness of prostates in the AST-treated rats was markedly lower than that in the BPH model control rats. It was also reported that the secretory granules that release their contents into the gland lumen increased in the T-induced BPH model as compared with the normal control [19,20]. In the present study, fewer secretory granules in the epithelia were observed in the AST-treated rats than in the BPH model control rats. These results indicate that AST might ameliorate the epithelial thicknesses of prostates and decrease the number of secretory granules in epithelia in T-induced BPH rats.

T and DHT are the principal androgens in the prostate, possibly related to the development of BPH [21]. The levels of T and DHT of prostates in the three AST-treated groups decreased as compared with the BPH model control group; a significant decline was found in the T level of prostates in the 40 mg/kg and 80 mg/kg AST-treated rats and the DHT level of prostates in the 40 mg/kg AST-treated rats, albeit not in a dose-response relationship. These results indicated that AST might cause the decrease in T and DHT levels in prostate tissues of T-induced BPH rats.

Oxidative stress, defined as the imbalance between the production and elimination of reactive oxygen species, can be alleviated by antioxidants and is one of several parameters considered to play a pivotal role in the development of BPH [22,23,24]. SOD, a group of antioxidant enzymes, plays an important role in oxidative stress in cells [25]. Research has suggested that erythrocyte SOD activity was significantly decreased in BPH patients as compared with age- and sex-matched healthy subjects [26]. Moreover, T injection in the BPH model has been shown to weaken cellular antioxidant mechanisms, including a decrease in SOD activity [27,28,29]. In this study, the SOD activity of prostates in the AST-treated rats increased, while the SOD activity of prostates in the 40 mg/kg and 80 mg/kg AST-treated rats was significantly higher than that in the BPH model control rats. These results demonstrated that AST might inhibit T-induced BPH in rats by regulating SOD activity. Further experiments on the mechanism by which AST regulates SOD activity in the present study will be carried out.

## 4. Materials and Methods

### 4.1. Materials

AST was purchased from Shanghai Aladdin Biochemical Technology Co., Ltd. (Shanghai, China) (lot no. F2030151). EPR was manufactured by Jiangsu Lianhuan Pharmaceutical Group Co., Ltd. (Jiangsu, China) (lot no. 20191002). Testosterone propionate was provided by Ningbo Second Hormone Factory (Ningbo, China) (lot no. 2003071). The SOD assay kit was obtained from Nanjing Jiancheng Bioengineering Institute (Nanjing, China) (lot no. 20210515). The T and DHT assay kits were purchased from Cusabio Biotech Co., Ltd. (Wuhan, China) (lot nos. C0307110304 and C0307100303).

### 4.2. Animals

The 72 male pathogen-free Sprague Dawley rats at 6–7 weeks of age were purchased from the Shanghai Sippr- BK Laboratory Animal Co., Ltd (Shanghai, China). Rats were housed 4 per cage with ad libitum access to water and feed in an environmentally controlled animal room (temperature 24 ± 2 °C, relative humidity 50 ± 10%, 12 h light-dark cycle) at the Shanghai Institute for Biomedical and Pharmaceutical Technologies. All experimental procedures were approved by the Animal Care and Use Committee of Shanghai Institute for Biomedical and Pharmaceutical Technologies (certificate no. 2020-29), and performed in conformity with the National Institutes of Health Guide for the Care and Use of Laboratory Animals.

### 4.3. Study Design and Treatment

After 5 days of acclimatization, the rats, except for twelve in the sham operation group, were castrated under pentobarbital sodium anesthesia before the experiment to prevent the influence of intrinsic testosterone. Except for the sham-operated rats which were just submitted to anesthesia and testicles exposure, castration was performed by removing testicles with epididymal fat via the scrotal route, as published previously [30].

Twelve normal rats, administrated olive oil orally and injected olive oil subcutaneously daily, served as the sham operation group. The other sixty castrated rats were randomly assigned into five groups (*n* = 12/group): (A) BPH model control group administrated olive oil orally and testosterone (5 mg/kg/day, s.c.), (B–D) AST groups treated with AST (20, 40 and 80 mg/kg /day, respectively) dissolved in olive oil orally and testosterone (5 mg/kg/day, s.c.), (E) EPR group (positive control group) given EPR (2 mg/kg/day) by oral gavage and testosterone (5 mg/kg/day, s.c.). EPR is used to treat benign prostatic hyperplasia to decrease prostate size and relieve the symptoms [31]. All rats in this experiment were treated once a day for four consecutive weeks. All animals’ body weights were measured once a week. At the end of experiment, under pentobarbital sodium anesthesia, prostates of all the rats were removed and weighed. One part of each ventral prostate lobe was fixed in 10% neutral buffered formalin for histopathological examination, while the remainder of each prostate was flash frozen in liquid nitrogen, and then stored at −80 °C for other analyses.

### 4.4. Prostate Index

The prostate index was calculated as the ratio of the prostate weight to the rat’s body weight.

### 4.5. Histopathological Examination

Following dehydration, the fixed prostates were embedded in paraffin, cut into 4 µm sections, and then stained with hematoxylin and eosin (H&E). Coverslips were mounted on the microscope slides containing tissue sections using mounting medium. Then, morphological changes were investigated and epithelium thicknesses of prostates were measured under a light microscope.

### 4.6. TEM

The ventral prostates were washed with normal saline solution and cut into pieces of about 1 mm^3^ on ice, and fixed with a 2.5% glutaraldehyde solution for 2 h at 4 °C. Following three washes with 0.1 M PBS, the tissues were postfixed in 1% osmium tetroxide for 2 h. After dehydration in ascending concentrations of ethanol and acetone, the tissues were embedded in Epon 618. Thin 70 nm sections stained with lead citrate were examined under a transmission electron microscope.

### 4.7. Analysis of SOD Activity

The frozen prostate tissue was homogenized in cold normal saline solution. The homogenate was centrifuged at 1520× *g* for 10 min at 4 °C. Then, the supernatants were collected, and the Bradford protein assay (Bradford assay) was used to quantify the protein amount. The SOD activity was assessed strictly according to the manufacturer’s protocol, as previously reported [32,33,34].

### 4.8. Analysis of Levels of T and DHT

The frozen prostate tissue was homogenized in cold normal saline solution. After two freeze-thaw cycles, the homogenate was centrifuged at 5000× *g* for 5 min at 4 °C. The supernatants were collected, while protein amounts were quantified by Bradford assay. Levels of T and DHT were assessed strictly according to the manufacturer’s protocol, as previously reported [35,36,37].

### 4.9. Statistical Analysis

Data obtained from this experiment are presented as means ± standard error of the mean (SEM). When equal variance was assumed, the data were evaluated using one-way analysis of variance (ANOVA) followed by post hoc LSD for multiple comparisons. When equal variance was not assumed, the data were analyzed by the non-parametric Kruskal– Wallis test with the Mann–Whitney U test for multiple comparisons. A significant difference was defined as *p* < 0.05 or *p* < 0.01.

## 5. Conclusions

The results of this study indicated that AST might have an inhibitory effect on T-induced BPH, especially in the ventral prostate, possibly due to the regulation of SOD activity and levels of T and DHT. Hence, AST may be a candidate as a novel therapeutic agent to inhibit BPH.

## Abbreviations

astaxanthin (AST); benign prostatic hyperplasia (BPH); dihydrotestosterone (DHT); epristeride (EPR); hematoxylin and eosin (H&E); superoxide dismutase (SOD); testosterone (T); transmission electron microscopy (TEM).
